# PLGA Microspheres of hGH of Preserved Native State Prepared Using a Self-Regulated Process

**DOI:** 10.3390/pharmaceutics12070683

**Published:** 2020-07-20

**Authors:** Jebun Nessa Diana, Ying Tao, Qiran Du, Meng Wang, Chinta Uday Kumar, Fei Wu, Tuo Jin

**Affiliations:** School of Pharmacy, Shanghai Jiao Tong University, 800 Dongchuan Road, Shanghai 200240, China; jebundiana@sjtu.edu.cn (J.N.D.); tina0715@163.com (Y.T.); Clytie_du@sjtu.edu.cn (Q.D.); Wangmeng_10086@sjtu.edu.cn (M.W.); cuday91@sjtu.edu.cn (C.U.K.); feiwu@sjtu.edu.cn (F.W.)

**Keywords:** recombinant human growth hormone (rhGH), sustained release microspheres, protein aggregation, stability

## Abstract

The challenges of formulating recombinant human growth hormone (rhGH) into sustained-release polymeric microspheres include two mutual causal factors, protein denaturing by the formulation process and severe initial burst release related with relative high dose. The stabilizers to protect the proteins must not evoke osmotic pressure inside the microspheres, and the contact of the protein with the interface between water and organic solution of the polymer must be minimized. To meet these criteria, rhGH was pre-formulated into polysaccharide particles via an aqueous–aqueous emulsion in the present study, followed by encapsulating the particles into microspheres through a self-regulated process to minimize the contact of the protein with the water–oil interface. Polysaccharides as the protein stabilizer did not evoke osmotic pressure as small sugar stabilizers, the cause of severe initial burst release. Reduced initial burst enabled reduced protein loading to 9% (from 22% of the once commercialized Nutropin depot), which in turn reduced the dosage form index from 80 to 8.7 and eased the initial burst. A series of physical chemical characterizations as well as biologic and pharmacokinetic assays confirmed that the present method is practically feasible for preparing microspheres of proteins.

## 1. Introduction

While sustained-release microspheres remain the only dosage form to date to reduce the injection frequency to once a month, and protein medicines which are given by injection are increasing, there is not yet a single protein drug formulated in this form. The only once marketed protein drug in this category, monthly-injecting human growth hormone (hGH) microspheres (Nutropin Depot), was dropped off from the market because of the complicated production process and severe burst release (C_max_ was nearly 80 times the effective blood concentration) [1]. To avoid protein denaturing, rhGH was precipitated with ZnCl_2_ prior to encapsulate in Nutropin depot by spraying the drug loaded polymer solution to liquid nitrogen. The ZnCl_2_ and liquid nitrogen resulted in severe burst due to osmotic pressure generated by the salt and porous structure formed in liquid nitrogen. Other extended release dosage forms of hGH such as hyaluronate microspheres, in situ thermal gelling polymer depot, and hGH microcrystals are free of organic solvents, but suffered from severe initial burst release too [2,3]. Absorbing proteins into pre-made porous polylactic-*co*-glycolic acid (PLGA) microspheres, followed by annealing to seal the pores, is another method to prepare sustained release microspheres without using organic solvent [4,5]. To enforce proteins to absorb into the pores, the microspheres were pre-loaded with alumina particles which improved protein encapsulation efficiency from 1% to 50% by the ability of the oxide to adsorb proteins. Since strong adsorption is known to cause protein denaturing, this method was limited to encapsulation of antigenic proteins as vaccines [6]. Although this microencapsulation method was reported for preparing hGH microspheres with considerable encapsulation efficiency and sustained release, the mechanism to ensue such good efficiency and its consequence for protein stability was not clarified [7].

While preparing microspheres by spray drying methods may prevent the proteins from contacting with the water–oil interfaces and the process may even be developed for continuous production [8,9,10], forming and hardening microspheres in such a short time may associate with reproducibility difficulties. Injecting microsphere-forming polymer solutions into a flowing continuous phase has been reported for preparing microspheres of desired quality, but a considerable number of the injecting nozzles and flow toughs may be required to achieve a practically feasible production efficiency [11]. The present study aimed to develop a practically feasible sustained-release microsphere dosage form of growth hormone without compromising the issues discussed above. The method of the present study to produce PLGA microspheres of hGH included two steps, pre-formulating the protein into dextran particles via an aqueous–aqueous emulsion [12], and encapsulating the particles in the microspheres using a self-regulated process [13]. The aqueous–aqueous emulsion allowed the protein to be partitioned preferentially in the dextran dispersed phase without contacting organic solvents and solidified by lyophilization hereafter to immobilize the native conformation of hGH. The process combined porous membrane-regulated formation, sedimentation-aided solidification, and container-guided collection of microspheres into a single unit operation, by which the contact of hGH with water–organic solvent interface (factor known to denature proteins) was minimized. This minimization also ensued 95%+ encapsulation efficiency of hGH, which in turn proved the minimized contact of the protein with water–organic solvent interface. The practical feasibility of this microencapsulation process in preparing hGH microspheres was examined in the present study through physical-chemical characterization and animal pharmacokinetics.

## 2. Materials and Methods

### 2.1. Materials

Poly(lactic-*co*-glycolic acid) (PLGA, acid terminated, lactide/glycolide molar ratio 50:50, and molecular weight 20 k and 30 k) were purchased from Evonik Corporation, Birmingham, AL, USA. Polyvinyl alcohol (PVA) (average molecular weight 9000–10,000, 80% hydrolyzed), dichloromethane, dextran (molecular weight was 70,000), Poly ethylene glycol (PEG) (average molecular weight was 20,000) were purchased from Sigma-Aldrich (St- Luise, MO, USA). rhGH was obtained from Shanghai United Cell Biotechnology Co. Ltd. (Chungchan, China), rhGH ELISA and IGF-1 ELISA kit were purchased from Mutlisciences (Lianke) Biotech. Co. Ltd. (Hangzhou, Zhejiang, China). Micro-BCA assay kit (Catalog No. 23235), microplate well (Product No. 15041), and cover plate using the Sealing Tape (Product No. 15036) were purchased from Thermo Fisher Scientific (Waltham, MA, USA). All the reagents and chemicals used in this study were of analytical grade.

### 2.2. In Vivo Rat Model

To examine the in-vivo release profile, approximately 200 g male Sprague Dawley (SD) rats and for the in-vivo efficacy another group of male Sprague Dawley rats (180–200 g, age 4–6 weeks) were purchased from Charles River Laboratory Co. Ltd. (Malvern, PA, USA) and for the animal studies the rats were maintained under standardized rodent condition at a room temperature of 22 ± 1 °C. All the animal experiments were approved by the institutional animal care and utilization committee of Shanghai Jiao Tong University complying the principles of laboratory animal care (Approval code: A2018018, approved on 20 March 2018).

### 2.3. Formulation of rhGH-Dextran Particles

A total of 3.5 mL of rhGH (0.55 %*w*/*v*), dextran (0.18 %*w*/*v*), and PEG (2.91 %*w*/*v*) were mixed in the ratio of 1:3:4. At first, dextran was dissolved in desalted rhGH solution and mixed thoroughly. PEG was dissolved in dextran-rhGH solution by stirring under magnetic stirrer until a clear solution was obtained. The solution was frozen overnight at −80 °C, followed by lyophilization for 48 h. The lyophilized particles were washed with 5 mL of acetonitrile followed by centrifugation at 8000 rpm for 10 min (Eppendorf 5415D, Hamburg, Germany) in order to remove the supernatant containing PEG in the continuous phase (the rhGH loaded dextran micro-particles could be collected at the bottom of the centrifuge tube as they are insoluble in acetonitrile). This washing-centrifugation method was repeated thrice and the final rhGH loaded dextran micro particles (rhGH-DMP) collected were dried to remove the dichloromethane residues.

### 2.4. Preparation of rhGH-DMP Loaded Microspheres by SPG Membrane Emulsion and Sedimentation Method

**Figure 1:**
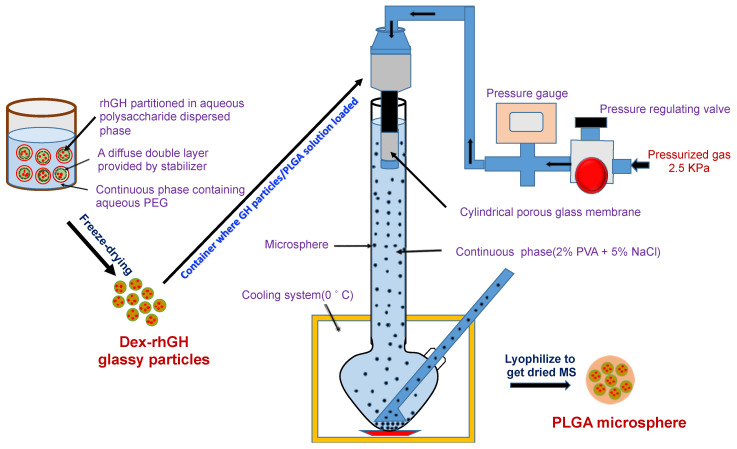
Schematic diagram of formulating dextran-recombinant human growth hormone (rhGH) micro particles loaded polylactic-*co*-glycolic acid (PLGA) microsphere.

Microspheres were prepared by using an apparatus (integrated microsphere preparation machine), which consist of a SPG membrane (20 µm pore size), an SPG emulsifying unit, a shaker, a pressure control system, a refrigeration unit, a glass column (3 L), and a microsphere washing and annealing unit (Figure 1) [12,13]. The initial pressure of nitrogen gas was about 12–15 MPa. At the beginning of the process the cooling system was turned on to keep the temperature of the continuous phase (2% PVA and 5% NaCl solution) around 0 °C. The dextran-rhGH micro particles were suspended in 16% PLGA solution in dichloromethane and homogenized at 2000 rpm for 3 min to get the particles comminuted and form a uniform suspension. This suspension was then transferred into a pressured container under 2.5 kPa and pressed through the porous membrane into the continuous phase (the PVA solution) to form embryonic microspheres (droplets of the PLGA solution). The embryonic microspheres of uniform size were allowed to freely fall to the bottom of the glass column containing 2% PVA and 5% NaCl solution at a slow and even rate and stored into the designed bottom. They were kept there for around 30 min to obtain hardened microsphere. The microsphere was washed with pre-cooled distilled water for several times to remove the salt and PVA and it was frozen following lyophilization to obtain dried microsphere before storage.

### 2.5. Scanning Electron Microscopy (SEM)

Scanning electron microscopy (SEM) of rhGH dextran micro particles and PLGA microspheres were observed by using a FEI Sirion 200 SEM (Hilboro, OR, USA). Before the image scanning, the samples were coated with gold particles at 5–10 keV under an argon atmosphere.

### 2.6. Particle Size Distribution of rhGH Loaded Dextran Micro Particles

To get high quality microspheres, the size and shape of rhGH loaded dextran particles should be considered. A particle size and shape analyzer (MICROTRAC S-3500, Osaka, Japan) was used to characterize the size, shape, and particle size distribution of rhGH dextran micro particles. A quartz cell with a stirring bar containing isopropyl alcohol and dry rhGH dextran particles (10 mg) were used in this experiment.

### 2.7. Encapsulation and Loading Efficiency of rhGH in Microspheres

A total of 5 mg of rhGH loaded microspheres was suspended in 2 mL of Dichloromethane and centrifuged at 8000 rpm for 25 min. This process was repeated three times to remove the PLGA from the microspheres completely leaving the rhGH dextran particles at the bottom of the tube. Prior to measuring the concentration by using a Micro BCA kit, the particles recovered by washing were dissolved in phosphate-buffer saline (pH 7.4).

### 2.8. Size Exclusion Chromatography

A HPLC system equipped with a TSK G2000SWXL size exclusion column (Shimadzu, Tokyo, Japan) was used to carry out the size exclusion chromatography. A peristaltic pump containing 50 mM phosphate-buffer saline (pH 7.3) was used to perform the elution. The flow rate was 1.0 mL/min at room temperature and the absorbance was measured at the wavelength of 214 nm. A 0.22 µm film was used to filter the samples and then injected into the HPLC instrument with a flow rate of the mobile phase adjusted to 1.0 mL/min. The size-exclusive high performance chromatography (SEC-HPLC) was used to identify if there was any formation of dimers or oligomers during the dextran-particles and microsphere forming process.

### 2.9. Circular Dichroism (CD)-Spectroscopy

The Jasco-JS 815 CD (Hachioji, Tokyo, Japan) Spectrometer was used and before transferring the rhGH containing samples into the sample cell, the sample cell was cleaned with pure water and dried naturally. Then the rhGH solution and other samples were transferred into the sample cell. For this test the wavelength for detection was 194–284 nm and the path length of fused quartz cells was 20 mm. The concentration of rhGH in the sample kept within 0.1–1 mg/mL. This test was carried out at room temperature and repeated thrice [11].

### 2.10. In Vitro Release Profile

A total of 20 mg of rhGH loaded PLGA microspheres was placed in a vial containing 2.0 mL of phosphate buffer saline (100 mM, pH 7.4) and incubated at 37 °C with constant shaking. On a schedule period of time the release medium was taken out for assay and replaced with fresh buffer. Micro-BCA assay kit was used to assay the rhGH content. For this experiment micro-plate Procedure (linear working range of 2.0–40 µg/mL) was used. Different dilutions of standard solution (0.5–200 µg/mL) and a blank sample were prepared. Then, 150 µL of standard, in vitro release samples, blank sample replicate, and working reagent were added into a micro-plate for this method and the plate was covered with the sealing tape. Then plate was incubated at 37 °C for 2 h. After cooling the plate to room temperature, the absorbance was measured at 562 nm on a plate reader. The concentration of the release samples was determined by preparing a standard curve (plotted the average blank-corrected 562 nm reading for each standard vs. its concentration in µg/mL). This experiment was repeated five times for each sample and the average of the repeat values was used to calculate the release profile.

### 2.11. In Vivo Release Study

Five groups of five male Sprague Dawley rat (6 weeks old and average 200 g/group) were injected subcutaneously with rhGH microspheres with three different doses 0.744, 2.23, and 2.98 mg/kg, blank microspheres, and native rhGH solution (0.07 mg/kg) of body weight of Dawley rat. An aqueous subcutaneous injection of microspheres was prepared with a vehicle consisting of 5.0 %*w*/*v* mannitol, 0.5 %*w*/*v* carboxymethyl cellulose, and 0.1 %*v*/*v* Tween 80. The time schedule for blood sample collection was set at 0, 0.5, 1, 2, 3, 4, 5, 6, 7, 8, 10, 12, 15 days after drug administration. A heparinized syringe was used to collect 0.4 mL blood from the tail vein. The plasma samples were collected by centrifuging the blood sample at 12,000 rpm at 4 °C for 20 min. An rhGH ELISA kit was used to determine the serum rhGH level.

### 2.12. In Vivo Efficacy Study

The rhGH microspheres, native rhGH solution, and blank microspheres with same dose mentioned above for in vivo release study were injected subcutaneously in 6-week-old Sprague Dawley rats for this study. The time schedule for blood sample collection was set at 0, 1, 2, 3, 4, 7, 14, 21, and 28 days after drug administration. A heparinized syringe was used to collect 0.4 mL blood from tail vein of each rat. An IGF-1 ELISA kit (Mutlisciences (Lianke) Biotech. Co. Ltd. (Hangzhou, Zhejiang, China) was used to determine the serum IGF-1 level.

### 2.13. Statistical Analysis

Statistically significant value was considered as *p* < 0.05. Mean ± standard deviation was used to express the data.

## 3. Results and Discussion

### 3.1. Physicochemical Properties of Dextran Particles and PLGA Microspheres

**Figure 2:**
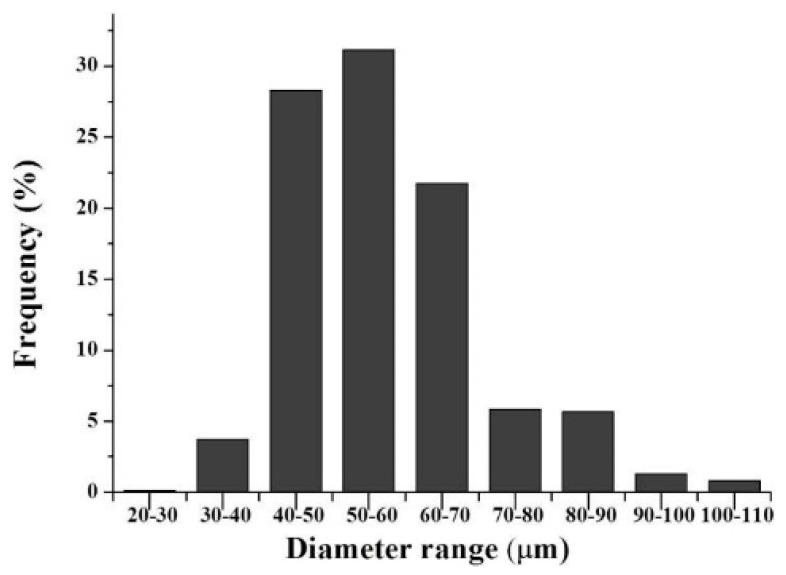
Size distribution diagram of the PLGA microspheres.

**Figure 3:**
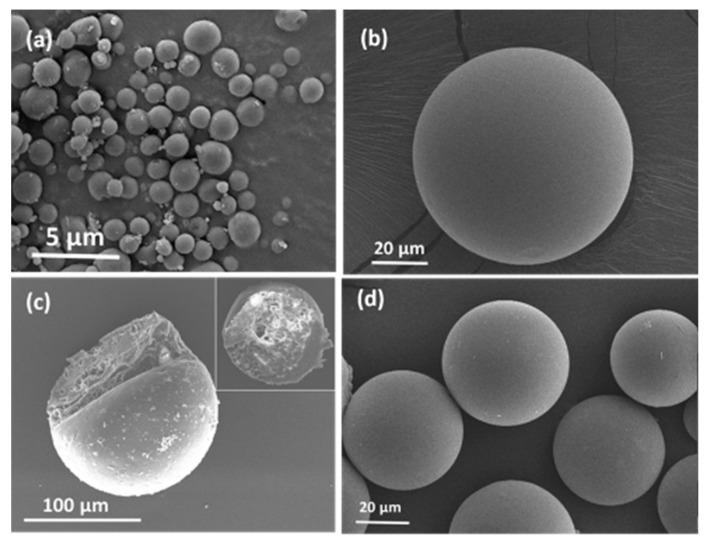
Scanning electron microscopy (SEM) images of (**a**) dextran-rhGH micro particles, (**b**) exterior image of a PLGA microsphere, (**c**) interior image of PLGA microsphere, and (**d**) rhGH loaded PLGA microspheres.

The morphology and size distribution of the rhGH-loaded micro particles and rhGH microspheres were characterized utilizing SEM (scanning electron microscopy) and dynamic laser scattering. The results confirmed the smooth surface and spherical shape of the two particles, as well as their respective diameter distributions centered at 1.96 µm with a polydispersity value 0.174 for the dextran particles and around 55.58 µm with standard deviation of 11.98 (Figure 2 and Figure 3) for the microspheres. The diameter ratio between rhGH-loaded dextran particles and PLGA microspheres is less than 1/20 which is believed to be appropriate for the so-called solid-in-oil-in-water (S/O/W) microencapsulation [14]. Moreover, the relatively uniform size of microspheres may help to reduce burst release due to under-sized small microspheres [15].

The rhGH encapsulation efficiency of the rhGH-dextran particles and microspheres was above 99% (*w*/*w*) and 95%, respectively. Our method for formulating rhGH-PLGA microspheres is a feasible and suitable approach to diminish severe burst release that could be robustly recommended by these results.

### 3.2. Conformational Stability of rhGH During the Microencapsulation Process

**Figure 4:**
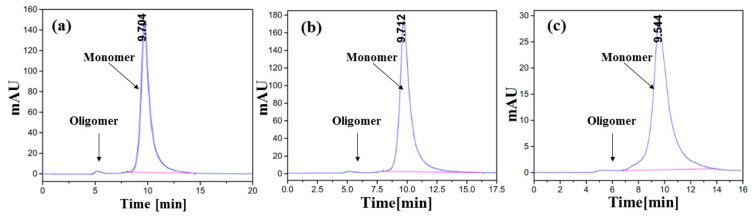
Size-exclusive high performance chromatography (SEC-HPLC) charts of (**a**) original rhGH solution, (**b**) dextran-rhGH particles, and (**c**) rhGH in PLGA microspheres.

Conformation changes including aggregation of proteins during microencapsulation processes often result in deactivation and even immunogenicity, and are thus regarded as a critical challenge in formulating proteins into microspheres [12]. To examine whether our protein stabilization strategy was effective, the encapsulated rhGH recovered from the dextran-rhGH particles and PLGA microspheres was characterized with the SEC-HPLC and circular dichroism (CD). As shown in Figure 4, the fresh rhGH and those recovered from the dextran particles and the final product, the PLGA microspheres displayed identical SEC-HPLC charts, indicating that our protected microencapsulation process did not cause aggregation of the protein.

**Figure 5:**
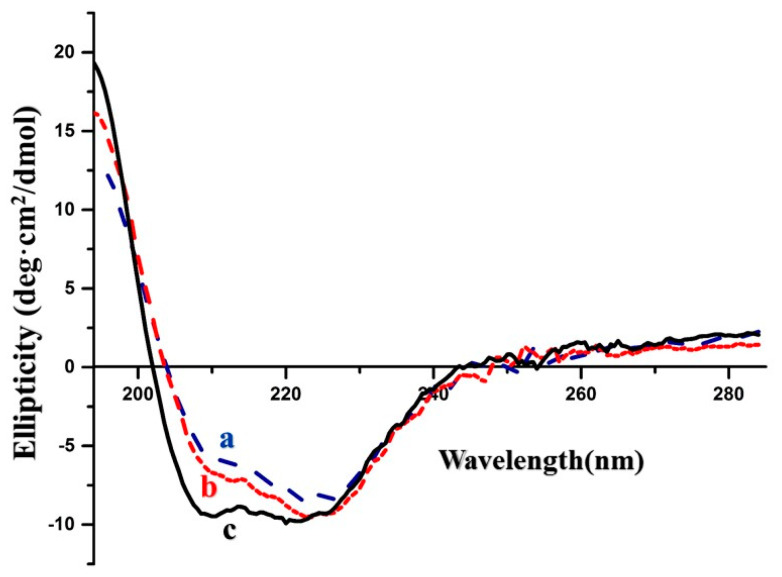
Circular dichroism spectra of (**a**) native rhGH solution, (**b**) dextran rhGH micro particles, (**c**) microsphere.

The conformation changes between the rhGH recovered from its original solution, the dextran micro particles, and the microspheres were examined using circular dichroism (CD), and the results are displayed in Figure 5. The slight differences between the curves representing the protein from the solution and the dextran particles from that from the PLGA microspheres may be attributed to the different α-helical content of 52.0%–59.8% of the formers and the latter, respectively. These differences comply with the variation range of the α-helical content of native rhGH, 55.0% ± 5% [16]. Taking the SEC-HPLC results into account together, we may speculate that the pre-formulation strategy prevented conformation changes of rhGH during the microencapsulation process involving organic solvent.

### 3.3. In Vitro Release Study

**Figure 6:**
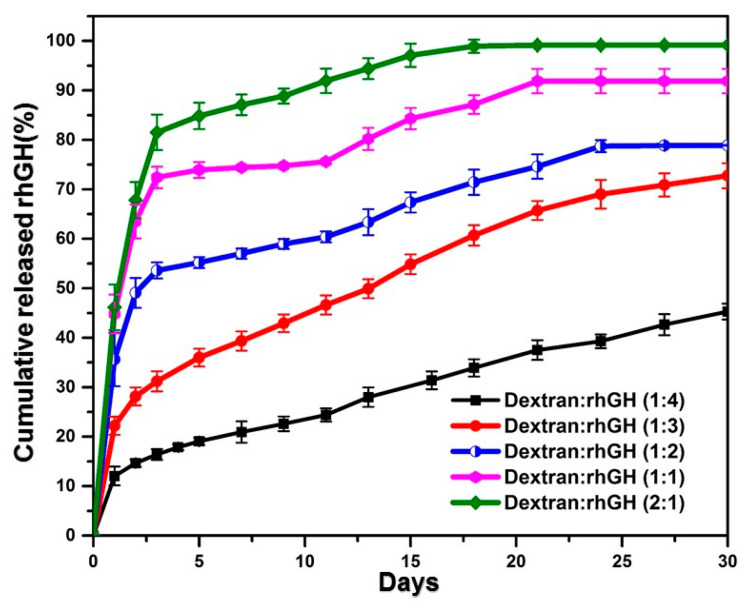
In vitro release profile of rhGH loaded PLGA microspheres with different dextran and rhGH ratios (*n* = 5, *p* < 0.05).

The release profile of rhGH from the PLGA microspheres was adjusted by varying the content of dextran for the expectation of the polysaccharide to create hydrophilic diffusion channels in the hydrophobic PLGA matrix [17]. The results of the in vitro release assays did show the expected relevance between rhGH release rate and dextran content that the protein released faster as the dextran content became higher (Figure 6). Selecting the content of dextran seems a tradeoff because raising the dextran content caused substantial initial burst, while reducing the content resulted in considerable incomplete release. We therefore chose the formulation wherein the protein load was 9% in mass and the dextran load was one third of the protein for successive pharmacokinetic (PK) study. To examine the effect of polymer molecular weight, the microspheres formed with the same drug/dextran/PLGA contents, but lower average molecular weight of the polymer was prepared for the PK study too.

### 3.4. Pharmacokinetic Studies in SD Rats

**Figure 7:**
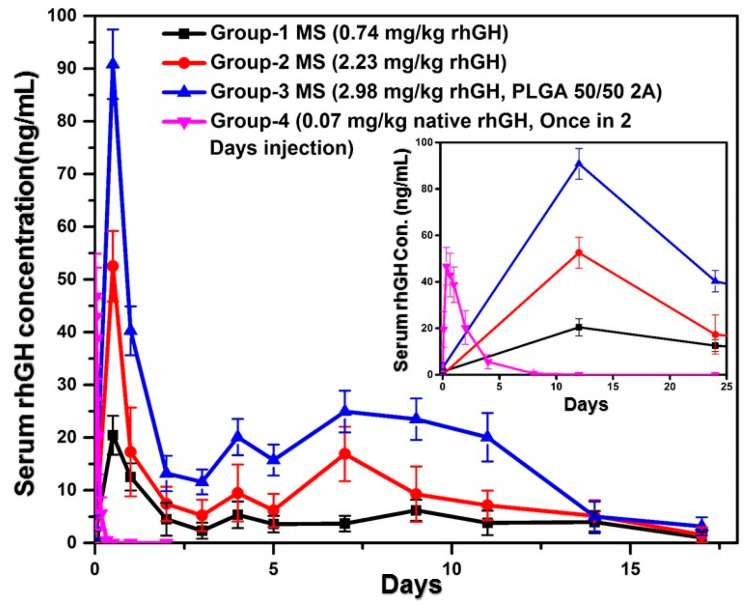
Serum concentration of rhGH after single subcutaneous injection of different treatments (*n* = 5, *p* < 0.05).

The selected microsphere formulation from the in vitro release assay was injected to three groups of SD rats subcutaneously with different doses and PLGA molecular weight, respectively. As a control, a group of rats that received rhGH solution was also included. Blood samples were taken at pre-determined time and assayed for rhGH concentration using ELISA. As summarized in Figure 7, the release profiles of rhGH from the microspheres consisted of a first day burst which was followed by a sustained release for two weeks. The rhGH release rate was in the order of microspheres of lower PLGA molecular weight (2A) > microsphere of higher PLGA molecular weight (2.5) of higher dose > microspheres of higher PLGA molecular weight of lower dose. Accordingly, the dosage form indexes (C_max_/effective concentration) were 28.66, 10, and 8.7, respectively. The rats that received the solution dosage form of rhGH showed a blood concentration profile of the initial burst only. By focusing on the first day kinetics, we found that the T_max_ of blood rhGH was 0.3 h for the solution dosage form but 12 h for the microspheres of the three dosage regimes (Figure 7).

Some related pharmacokinetics parameters were determined from the blood concentration profiles and summarized in Table 1. At the T_max_ of 0.3 h, the C_max_ of the once in 2 days injection reached 46.71 ± 26.72 ng/mL but after 8 h of administration, it declined to the baseline (Figure 7). Among the three microsphere groups that contains PLGA 50/50 2A exhibited the highest C_max_ 90.82 ± 8.01 ng/mL at the Tmax of the first sampling point (12 h), and it also exalted the plasma concentration of rhGH more than 14 days after administration. The groups administered with the dose of 0.744 and 2.32 mg/kg reached the C_max_ of 20.43 ± 4.99 ng/mL and 52.52 ± 8.44 ng/mL, respectively. All the doses exhibited minimum early burst. The burst releases for different doses were determined by the ratio of AUC_(0–1d)_/AUC_(0–14d)_ [18].

### 3.5. Efficacy Assays of rhGH Microspheres

**Figure 8:**
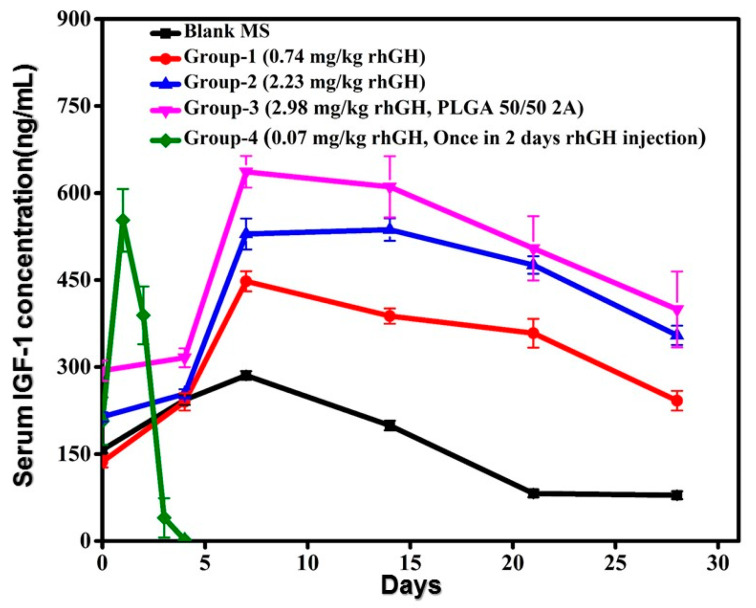
Serum concentration of IGF-I after single subcutaneous injection of different treatments (*n* = 5, *p* < 0.05).

The efficacy of rhGH microspheres was examined by measuring the changes of insulin like growth factor (IGF-1), as IGF-1 increases along with rhGH concentration which was induced by the injected rhGH in the animals. Figure 8 exhibits the IGF-1 release profile up to 28 days on the three groups of animals. After the subcutaneous injections all the groups having different doses achieved a stable and sustained profile for 28 days. The serum IGF-1 concentration elevated depending on the dose administered. The *C_max_* of the formulation group-1, group-2, and group-3 was 344.97 ± 102.89 ng/mL, 445.85 ± 91.11 ng/mL, and 518.12 ± 118.58 ng/mL, respectively. Whereas, for rhGH solution the *C_max_* was 379.89 ± 173.29 ng/mL. The concentration of IGF-1 in rhGH solution (group-4) showed a rapid fall to the baseline by day 4, but the other groups sustained up to day 28. It is evident from these results that it is possible to achieve a sustained release profile of IGF-1 with the rhGH microspheres prepared by this method.

Sustained release is a complicated process. After long and extensive study, it has been accepted that the release kinetics of controlled release microspheres is actually not dominated by zero-order release kinetics but three other mechanisms [19]. The major mechanisms are (1) diffusion-sustained release through polymer matrix, (2) hydration-sustained release because of swelling of polymers, formation of new pores and osmotic pumping, (3) erosion of the PLGA matrix or a combination of all the three mechanisms in a different step. Loading proteins in polysaccharide fine particles before microencapsulation may be a practical approach as the dextran particles during the controlled release can be retained longer than proteins in the PLGA microspheres matrix [20]. However, this process has two effects at the same time, while reducing the incomplete release yet it may also cause burst release [21]. Burst release of protein also connected to the amount of protein stabilizers blended in PLGA microspheres, so by selecting an appropriate amount of stabilizer it is assumed that protein can be protected effectively during the total controlled release period while reducing early burst release. Therefore, in this study a different amount of protein stabilizers (dextran) was used to formulate dextran rhGH micro particles and the ratio of dextran and rhGH (1:3) was shown as the most feasible one. Moreover, water soluble proteins are thermodynamically unstable in concentrated PEG solution and better to protect with dextran particles within a PLGA matrix [12,22].

PLGA matrix degradation may generate acid formation which could accumulate inside the microsphere [23,24]. When a sustained release polymer matrix contains dispersed protein particles inside it with a low loading, it could be considered that every protein particle is a separated dot and the main mechanism for the drug release is diffusion through the polymer matrix. The drug particles begin to connect each other with the increment of drug loading which start forming diffusion channels at hydrous state [25,26]. While, PLGA degradation due to surface erosion at first holes or pores on the surface have been created which generates adequate penetration of fluid diffusion channels for the protein release from the microspheres. Adsorption of fluid motivates swelling in the dextran particles which decreases protein diffusion distance from inner to outer microspheres as compared to additives without swelling properties [27]. This study also alludes to the fact that initially when the release phase begins rhGH was close to the coating periphery of the microsphere, more and more pores started to form at the internal part of the microsphere with the beginning of PLGA degradation. As a result, water penetrated into the bulk core structure of the microspheres and dissolved the dextran particles. rhGH release from the microspheres prepared by this method could be attributable to the mechanism containing surface erosion and bulk degradation [17]. The process that combined porous membrane-regulated formation, sedimentation-aided solidification, and container-guided collection of microspheres into a single unit operation can be utilized to achieve a high loading encapsulation with low early burst release, and feasible release kinetics. The simplified production process may turn microspheres from a complicated dosage form to a popular one.

## 4. Conclusions

By pre-formulating rhGH into dextran particles via an aqueous–aqueous emulsion/phase with precisely optimized protein/dextran ratio, rhGH may be encapsulated in PLGA microspheres with preserved native state and released in a sustained profile with reduced dosage form index. The self-regulated microencapsulation process which combined porous membrane-regulated formation, sedimentation-aided solidification, and container shape guided collection of microspheres further minimized the contact of the protein with the water–oil interfaces. The method of this report for formulating microspheres may offer a practically feasible solution for producing sustained release microspheres of protein medicines.

## Figures and Tables

**Table 1:** Pharmakokinetic studies on group-1 (0.744 mg/kg rhGH), group-2 (2.23 mg/kg rhGH), group-3 (2.98 mg/kg rhGH, PLGA 50/50 2A), and group-4 (0.07 mg/kg native rhGH, once in 2 days injection).

Pharmacokinetic Parameters	Group-1	Group-2	Group-3	Group-4
	(0.744 mg/kg rhGH)	(2.23 mg/kg rhGH)	(2.98 mg/kg rhGH, PLGA 50/50 2A)	(Once in 2 Days Injection)
Tmax (day)	0.5	0.5	0.5	0.0139 (0.3 h)
Cmax (ng/mL)	20.43 ± 4.99	52.52 ± 8.44	90.82 ± 8.01	46.71 ± 26.72
AUC (0–1 d) (ng·day/mL)	22.21 ± 5.15	42.92	82.95 ± 6.22	4.44 ± 1.89
AUC (0–2 d) (ng·day/mL)	28.88 ±7.84	48.46 ± 2.34	81.63 ± 6.24	4.44 ± 1.89
AUC (0–14 d) (ng.day/mL)	80.15 ± 22.61	158.48 ± 53.05	311.59 ± 48.89	-
Early burst (%)	27.71	27.09	26.62	-
DI (dosage form index) (0–2 d)	4.54	7.09	6.9	85.19
